# Biological Activities and Chemical Compositions of *Prangos platychlaena* Boiss. Essential Oil and Fractions, Multivariate Statistical Analyses

**DOI:** 10.1021/acsomega.3c05700

**Published:** 2024-02-29

**Authors:** Damla Kırcı, Ceyda Sibel Kılıç, Hayri Duman, Betül Demirci

**Affiliations:** † Department of Pharmacognosy, Faculty of Pharmacy, Selçuk University, Konya 42000, Türkiye; ‡ Department of Pharmaceutical Botany, Faculty of Pharmacy, Ankara University, Ankara 06560, Türkiye; § Department of Biology, Faculty of Science, Gazi University, Ankara 06560, Türkiye; ∥ Department of Pharmacognosy, Faculty of Pharmacy, Anadolu University, Eskişehir 26000, Türkiye

## Abstract

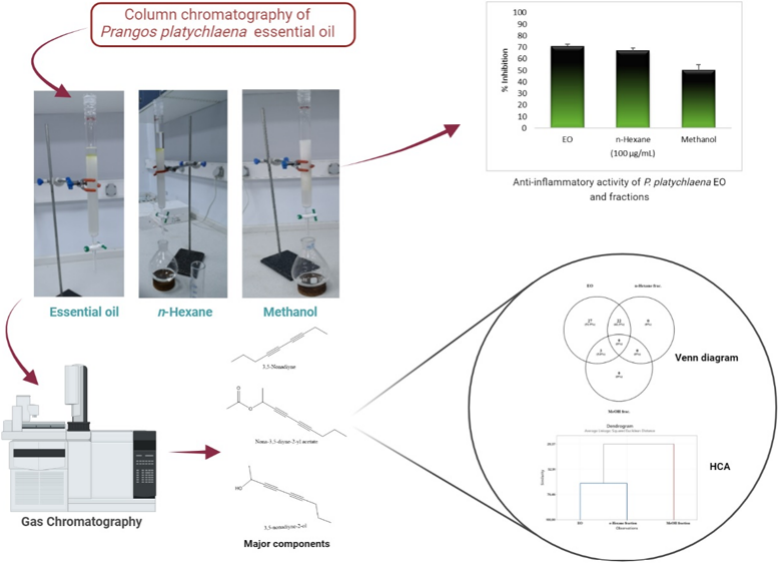

In this research, essential oil was obtained from the aerial parts of *Prangos platychlaena* Boiss. by hydrodistillation using a Clevenger-type apparatus, separated into fractions having different polarities by column chromatography. Both essential oil and the fractions were analyzed by GC-FID and GC/MS simultaneously. Nona-3,5-diyne-2-yl acetate (46%) and 3,5-nonadiyne (13.5%) were found to be the main constituents of the essential oil. While the main components of the *n-*hexane fraction were characterized as 3,5-nonadiyne (45.6%) and germacrene B (16.4%), the major components of the methanol fraction were found to be nona-3,5-diyne-2-yl acetate (59.6%) and 3,5-nonadiyne-2-ol (25.9%). In addition, principal multivariate statistical analyses were performed with principal component analyses and Venn diagram calculations, utilizing chemical compositions of the essential oil and the fractions. Furthermore, *in vitro* anti-inflammatory activities of the essential oil and the fractions were evaluated to correlate the chemical composition with the biological activity, and to the best of our knowledge, this study was performed for the first time in this aspect. LOX inhibitions of the essential oil, *n-*hexane, and methanol fractions were determined to be 70.98 ± 1.7, 67.10 ± 2.5, and 50.11 ± 4.8%, respectively. Preliminary initial findings of this study will be extended in the future with new biological assays.

## 1 Introduction

Members of the Apiaceae family are important sources with respect to the pharmaceutical and agrochemical industries. The Apiaceae family is represented by 511 taxa in Turkey, with approximately 485 species and 181 endemic taxa present among them. *Prangos* L. is a perennial genus of the Apiaceae family, with 30 species having worldwide distribution. The genus is known for its winged fruits, and its diversity center is in the Irano-Turanian phytogeographic area; thus, it is commonly called “Jashir” in Persian.^1,2^

*Prangos* species have various uses in traditional medicine and food flavoring in Iran. They have been reported to possess emollient, tonic, carminative, antispasmodic, and antihemorrhoidal properties and have the ability to stop external bleeding and promote scar healing as well. *Prangos* species also exhibit cytokine release-inhibiting capacity and antibacterial, antioxidant, antifungal, insecticidal, and anti-HIV activities. These findings suggest that *Prangos* species may have potential applications in the food and pharmaceutical industries.^2−4^

Previous studies carried out on the phytochemistry of *Prangos* species have identified various natural compounds, such as terpenoids, flavonoids, alkaloids, and coumarins, which have been isolated from different parts of the plants. Medicinal properties of *Prangos* species are attributed to their secondary metabolites, particularly to the essential oil (EO) of the fruits. Studies have shown that EO compositions of various *Prangos* species are dominated by monoterpene hydrocarbons.^2,5^

*Prangos platychlaena* Boiss. is a perennial herbaceous plant, which is endemic for Türkiye and is known locally as çağşır, çakşır, korkor, and kirkor.^4,6^ Both the fruits and roots of this plant are recognized for their potential medicinal properties. *P. platychlaena* is traditionally used to stop bleeding in the skin or to heal scars in eastern Türkiye externally.^2,3^

There is limited information available regarding the EO composition of *P. platychlaena*, a medicinal plant growing in the mountainous regions of Iran, Iraq, and Türkiye. Studies have revealed that EOs of *P. platychlaena* from different regions contain various compounds, including α-pinene, β-phellandrene, δ-3-carene, and *p*-cymene.^6,7^

Within the scope of this work, the EO and fractions of this EO were evaluated comparatively in terms of their biological activities and chemical compositions. Hydrodistillation was utilized to obtain EO using a Clevenger-type apparatus for a period of 3 h. EO then was separated into fractions with solvents having different polarities using column chromatography. EO and the particles were analyzed by GC-FID and GC/MS simultaneously. Their chemical compositions were analyzed with the statistical method, in which hierarchical cluster analysis (HCA) and the Venn diagram were used as tools. Moreover, *in vitro* anti-inflammatory activities of the EO and the fractions were evaluated for the correlation of phytochemical composition with the biological activity, which has been performed for the first time in this respect as far as we are concerned.

## 2 Results and Discussion

### 2.1 GC-FID and GC/MS Analyses

Fifty-one compounds were identified within the composition of the EO, including 14 monoterpene hydrocarbons, 7 oxygenated monoterpenes, 14 sesquiterpene hydrocarbons, 5 oxygenated sesquiterpenes, 1 fatty acid, and 10 others. 3,5-Nonadiyne and 3,5-nonadiyne derivatives are alkyne compounds found in the EO that were included in the group specified as others (67.5%). Nona-3,5-diyne-2-yl acetate (46%) and 3,5-nonadiyne (13.5%) were found to be the main constituents of *P. platychlaena* EO. Compositions of the EO and the fractions are listed in Table 1.

**Table 1:** Chemical Compositions (%) of *P. platychlaena* EO and the Fractions

**KI**^a^	**RRI**^b^	**compound**	**EO**	*n***-hexane fraction**	**methanol fraction**
1025^11^	1032	α-pinene	3.5	0.3	-
1026^11^	1035	α-thujene	0.1	-	-
1040 ± 35^12^	1048	2-methyl-3-buten-2-ol	0.2	-	-
1068^11^	1076	camphene	0.2	-	-
1092^13^	1093	hexanal	tr	-	-
1110^11^	1118	β-pinene	0.3	-	-
1122^11^	1132	sabinene	0.2	-	-
1160^11^	1174	myrcene	0.3	-	-
1167^11^	1176	α-phellandrene	1.4	0.5	-
1198^11^	1203	limonene	0.8	0.7	-
1209^11^	1218	β-phellandrene	6.2	5.8	-
1232^11^	1244	2-pentyl furane	tr	-	-
1245^11^	1255	γ-terpinene	0.1	-	-
1273^11^	1266	(*E*)-β-ocimene	0.3	0.2	-
1270^11^	1280	*p*-cymene	4.5	5.9	-
1282^11^	1290	terpinolene	1.0	1.5	-
	1327	3-Methyl-2-buten-1-ol	0.1	-	-
	1415	3,5-nonadiyne	**13.5**	**45.6**	-
	1452	α*,p*-dimethylstyrene	tr	-	-
1487^11^	1495	bicycloelemene	tr	-	-
	1473	(*Z*)*-*3,5-nonadiyne-7-ene	0.5	1.6	-
	1539	(*E*)*-*3,5-nonadiyne-7-ene	0.3	1.4	-
1561^11^	1582	*cis*-chrysanthenyl acetate	tr	0.1	-
1579^11^	1590	bornyl acetate	0.2	-	-
1590^11^	1600	β-elemene	0.1	0.7	-
1598^11^	1612	β-caryophyllene	tr	-	-
1614^11^	1638	*cis-p-*menth-2-en-1-ol	tr	-	-
1639^11^	1650	γ-elemene	0.1	0.4	-
1651^11^	1668	(*Z*)-β-farnesene	tr	-	-
1666^11^	1687	α-humulene	0.2	1.7	-
	1688	selina-4,11-diene	tr	0.7	-
1674^11^	1690	cryptone	0.1	-	-
1708^11^	1726	germacrene D	0.4	2.8	-
1728^11^	1740	valencene	0.1	1.4	-
1734^11^	1755	bicyclogermacrene	0.5	2.4	-
1755^11^	1773	δ-cadinene	0.1	0.9	-
1763^11^	1776	γ- cadinene	tr	0.2	-
1764^11^	1785	7-*epi*-α-selinene	0.1	1.0	-
1784^11^	1802	cumin aldehyde	tr	-	-
1823^11^	1854	germacrene B	**1.7**	**16.4**	-
1848^11^	1864	*p*-cymen-8-ol	tr	-	1.7
1868^11^	1878	2,5-dimethoxy-*p*-cymene	tr	-	-
	1903	nona-3,5-diyne-2-yl acetate (isomer)	3.8	-	-
	1915	nona-3,5-diyne-2-yl acetate	**46.0**	-	**59.6**
2126^11^	2144	spathulenol	0.1	-	-
2165^12^	2178	*T*-cadinol	0.1	-	-
2069^11^	2202	germacrene *D*-4-ol	0.1	-	-
	2191	3,5-nonadiyne-2-ol	**3.1**	-	**25.9**
	2209	*T*-muurolol	0.1	-	-
2227^11^	2255	α-cadinol	0.2	-	-
2913^11^	2931	hexadecanoic acid	0.2	-	-
		monoterpene hydrocarbons	**18.9**	**14.9**	-
		oxygenated monoterpenes	0.3	0.1	1.7
		sesquiterpene hydrocarbons	**3.3**	**28.6**	-
		oxygenated sesquiterpenes	0.6	-	-
		fatty acid	0.2	-	-
		others	**67.5**	**48.6**	**85.5**
		**total**	**90.8**	**92.2**	**87.2**

a KI: from literature.^11−13^

b RRI: relative retention indices calculated against *n*-alkanes (C7–C40). %, relative percentage calculated from FID data; tr, trace (<0.1%); -, not detected.

EO (500 mg) was separated into fractions with different polarities, i.e., *n*-hexane and methanol, respectively, to yield 98 mg (19.5%) *n*-hexane and 400 mg (80%) methanol fractions. The *n*-hexane fraction was characterized as having 3,5-nonadiyne (45.6%) and germacrene B (16.4%) as the main components, while the main components of the methanol fraction were determined to be nona-3,5-diyne-2-yl acetate (59.6%) and 3,5-nonadiyne-2-ol (25.9%).

When we performed literature search related to the composition of EO of *P. platychlaena* fruits, we found that a study performed on the plant growing in the southeast of Türkiye (Hakkari) existed. Although the authors did not specify the subspecies of *P. platychlaena*, major compounds including α-pinene (69.8%), β*-*phellandrene (10.6%), δ-3-carene (3.4%), and *p-*cymene (3.4%) were reported.^8^ The present EO appears to have more complex and varied composition than the EO previously described.^7^ In another study, EOs of *P. platychlaena* fruits collected from two different cities of Türkiye, namely, Malatya and Sivas, were characterized by a series of acetylenic derivatives like 3,5-nonadiyne (24.5 and 5.8% in the EO of fruits from Malatya and Sivas species, respectively), (*Z*)*-*3,5-nonadiyne-7-ene (0.2% in Malatya), and (*E*)*-*3,5-nonadiyne-7-ene (0.5% in Malatya).^6^ 3,5-Nonadiyne was identified in the rhizomes of *Cachrys ferulacea* L. and roots of *Selinum tenuifolium* Wall ex C.B. Clarke that also belong to the Apiaceae family.^9^

Another study related to the EO composition^2^ was performed in southwestern Iran, and the EO yields of the aerial parts of different *P. platychlaena* populations varied from 0.4 to 2.85% (v/w). The main components found in the EO of the aerial parts were α-pinene, β-pinene, *D*-3-carene, β-phellandrene, α-terpinolene, α-thujone, and β-caryophyllene. However, the percentages of each component in these EOs showed variations according to 13 different habitats that the specimens were collected from.^2^ In contrast, the EO of *P. platychlaena* and EOs of *Prangos* species in general are dominated by monoterpene hydrocarbons.^10^

### 2.2 Multivariate Statistical Analyses

**Figure 1:**
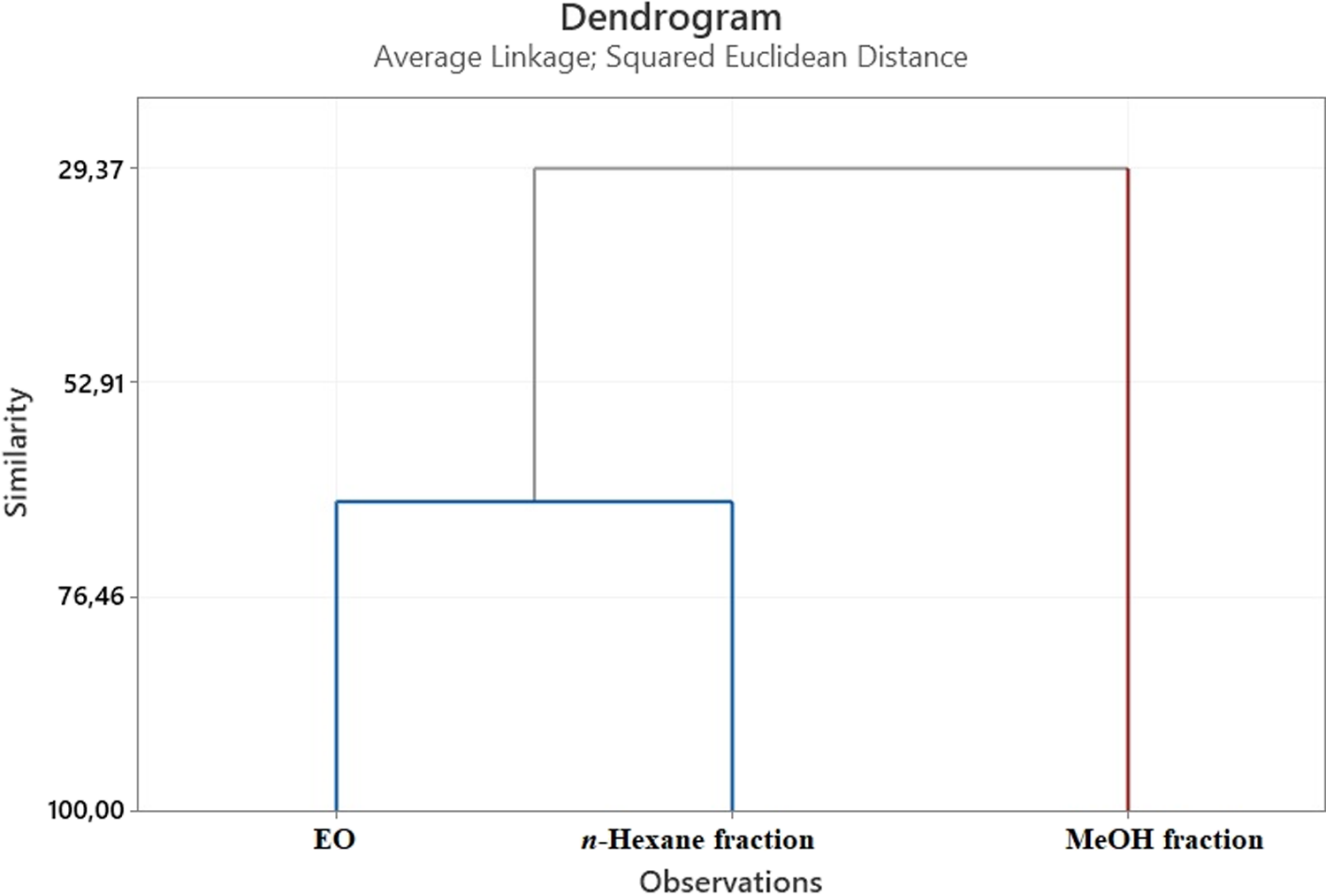
Dendrogram obtained by HCA based on Euclidean distances between groups of the major compounds of EO and the fractions.

Statistical HCA and the Venn diagram demonstrated variations in the composition of EO and the obtained fractions. HCA was performed on the EO, factions, and also six major components that we have determined to be present in this study (β-phellandrene, *p*-cymene, germacrene B, 3,5-nonadiyne, nona-3,5-diyne-2-yl acetate, and 3,5-nonadiyne-2-ol) using Minitab 19 software. The cluster analysis of EO and the fractions (Figure 1) revealed two main clades, with similarity ranging from 29.37 to 66.50%. The HCA similarity of the EO and *n*-hexane fractions was 66.50%.

**Figure 2:**
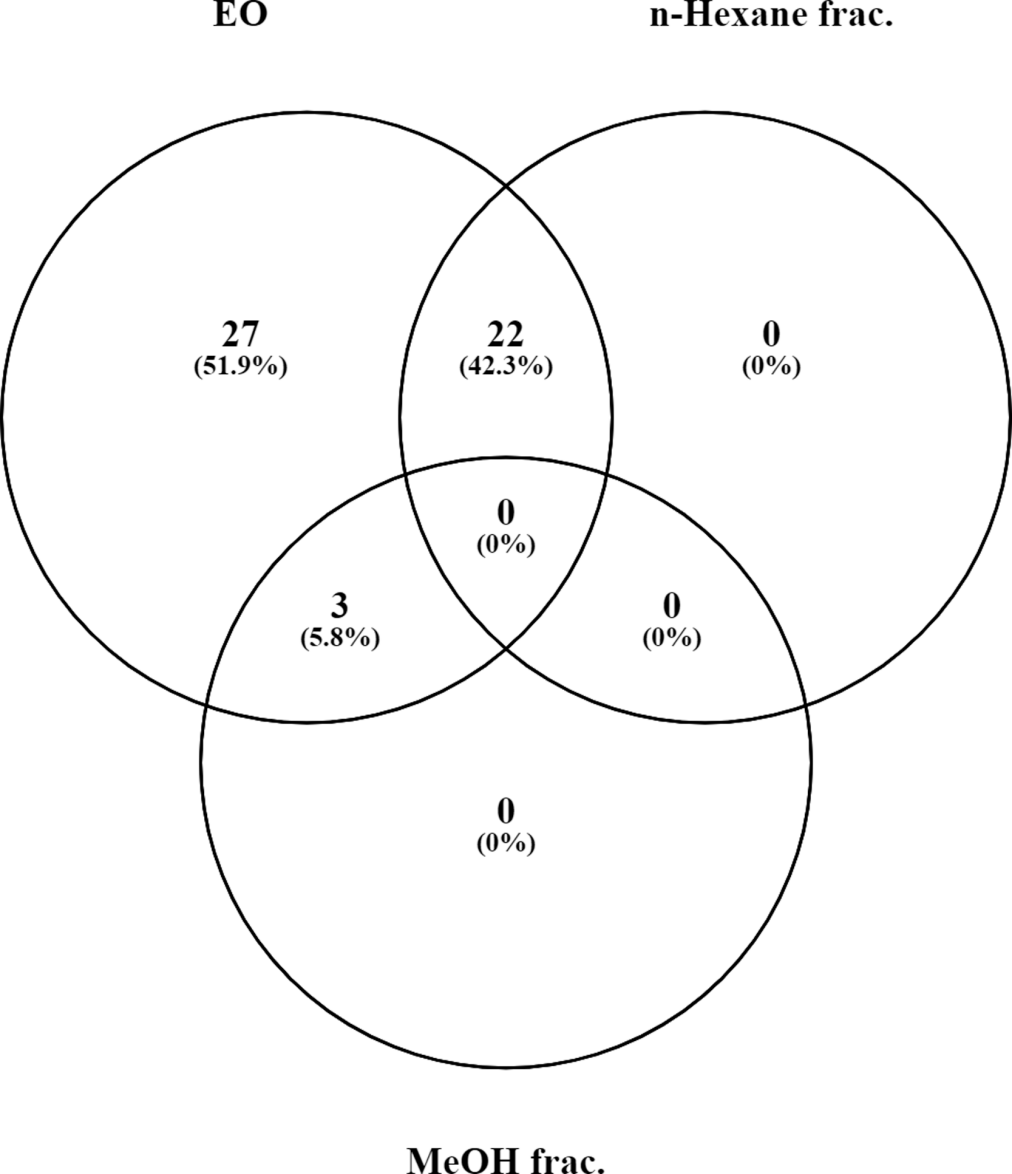
Venn diagram of EO and the fractions.

Any difference in the presence of the identified components in the EO and the fractions was determined using a Venn diagram (Figure 2). The EO was the richest sample with respect to the chemical composition. For instance, while 52 compounds were identified in the EO, the three components (3,5-nonadiyne-2-ol, nona-3,5-diyne-2-yl acetate, and *p*-cymen-8-ol) were found to be present in the methanol fraction.

### 2.3 5-LOX Inhibitory Activity

The 5-lipoxygenase (5-LOX) enzyme plays a crucial role in the production of leukotrienes, which are proinflammatory lipid mediators derived from arachidonic acid. Leukotrienes have been implicated in various inflammatory diseases, such as asthma and atherosclerosis. Additionally, emerging evidence suggests that 5-LOX metabolites may affect tumorigenesis, linking them to their potential implications in cancer. Understanding the biochemistry of this enzyme might have significant implications in the treatment of various diseases, as it may offer opportunities in the development of therapeutic interventions targeting the production of leukotrienes and their associated inflammatory pathways.^14^

**Figure 3:**
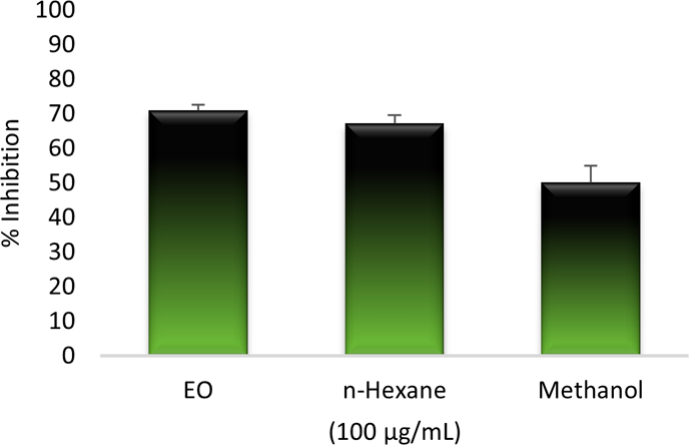
Anti-inflammatory activity of *P. platychlaena* EO and fractions.

In this study, *in vitro* anti-inflammatory activity was evaluated by the (5-LOX) inhibitory effect of the EO and the fractions spectrophotometrically, while NDGA was used as the positive control. The IC_50_ value was found to be 3.63 ± 0.29 μg/mL, while the positive control was calculated to be 100 ± 0% in 100 μg/mL. The anti-inflammatory activities of the EO, *n*-hexane, and methanol fractions were determined to be 70.98 ± 1.7, 67.10 ± 2.5, and 50.11 ± 4.8% in 100 μg/mL, respectively (Figure 3). To the best of our knowledge, this is the first report on the enzyme inhibitory activity of EO in the fruits of *P. platychlaena*.

Previously, researchers investigated the anti-inflammatory and anticarcinogenic activities of methanolic and aqueous extracts of *P. platychloena* on colorectal cancer cell lines CCL-221 and Caco-2. The trypan blue exclusion test was employed to monitor the aforementioned activities. Regarding the anti-inflammatory effects, researchers investigated the extracts’ impact on the secretion of interleukin 8 (IL-8) and interleukin 6 (IL-6) in cancer cells, induced by tumor necrosis factor-α. At a concentration of 1000 μg/mL, the aqueous extract significantly decreased IL-8 secretion from 519.07 to 28.3 pg/mL in CCL-221 and IL-6 secretion from 63 to 1 pg/mL in CCL-221. The methanol extract also showed anti-inflammatory properties, however to a lesser extent.^15^

In another study, researchers isolated four coumarins from the extracts prepared from *P. haussknechtii* Boiss. The inhibitory activity of the four compounds (coumarins 1, 2, 3, and 4) was tested on the COX-1 and COX-2 enzymes. Coumarins 1 and 2 showed significant COX-1 enzyme inhibitory activity, with IC_50_ values of 36.8 and 47.7 μM, respectively, comparable to those of over-the-counter nonsteroidal anti-inflammatory drugs aspirin, ibuprofen, and naproxen. Furthermore, coumarin 4 specifically inhibited COX-2 enzyme with an IC_50_ value of 34.6 μM, similar to the prescription anti-inflammatory drug Celebrex.^14,16^

Some studies related to the anti-inflammatory activities of compounds isolated from the extracts of *Prangos* species are also found in the literature, reporting significantly high biological activities. Our study and works of literature show that both volatile and nonvolatile compounds/extracts of *P. platychlaena* have anti-inflammatory effects with a selective or dual by inhibiting the arachidonic acid pathway. In this study, anti-inflammatory activity was found to be high for the EO, and thus, we can say that *Prangos* species have the potential to treat inflammatory diseases.

The composition of the EO, the fractions, and the chemical variability by using HCA analysis were examined. The results of the multivariate statistical studies (HCA and Venn diagram), the chemical compositions of EO, and the methanol and *n-*hexane fractions were compared with chemotaxonomic information determined with our research and with literature studies. Studies of the *Prangos* species were generally conducted on the stem, leaves, and flowers. However, the composition of the fruits was investigated, a significant contribution to the literature.

EOs are generally known for their antimicrobial effects. Anti-inflammatory activity may not be present in every EO; therefore, this study is critical. Additionally, the anti-inflammatory activities of both the EO and fractions were observed in the enzyme study. To the best of our knowledge, this is the first report on the enzyme inhibitory activity of EO of fruits of *P. platychlaena*. While the EO had a similar chemical composition compared with the studies found in the literature, the chemical composition that was found to be closest to the EO belonged to the *n*-hexane fraction. While 3,5-nonadiyne was identified as the main compound of both EO and *n*-hexane fraction, nona-3,5-diyne-2-yl acetate was the other main compound that was present in both the EO and the methanol fraction (59.6%). These two compounds were considered to be affected in respect to the bioactivity of the plant. In conclusion, the activity of the EO, which contains these two major components, was higher than the obtained fractions, probably demonstrating a synergistic effect. Based on these findings, we can conclude that the EO oil of the fruits of *P. platychlaena* could be a promising candidate for pharmaceutical applications and conventional drugs due to its remarkable anti-inflammatory activity on 5-LOX.

## 3 Methods

### 3.1 Plant Material

*P. platychlaena* was collected in 2018 from Cimil Mountain, Erzincan, Türkiye (39° 42.127′N/39° 45.874′W). The plant material was identified by Prof. Dr. Hayri Duman from Gazi University Faculty of Science, Department of Biology, and was deposited at the Herbarium of the Faculty of Pharmacy of Medipol University in Istanbul, Türkiye (Herbarium code: IMEF 2354).

### 3.2 Isolation of Essential Oil and Fractionation

EO of *P. platychlaena* fruits (100 g) was obtained by hydrodistillation using a Clevenger-type apparatus for 3 h. The yield of EO was 0.79% (w/w).

EO fractionation was performed with manual column chromatography.^14^ Silica gel 60 (Meck-7734, 0.06–0.2 mm) was used as the chromatographic adsorbent. Silica gel was allowed to activate for 2 h at 100 °C. After that, it was mixed with *n*-hexane and then introduced into the column at room temperature. The obtained EO (500 mg) was loaded into the column using *n-*hexane. EO was separated into fractions with solvents having different polarities and yielded *n*-hexane (98 mg) and methanol (400 mg) fractions.

### 3.3 GC-FID and GC/MS Analysis

EO of *P. platychlaena* fruits and the fractions were analyzed by GC-FID and GC/MS using an Agilent GC-mass selective detector (MSD) system. GC/MS analyses were carried out with an Agilent 5975 GC/MSD system. An Innowax fused silica capillary column was used with helium as the carrier gas. The oven temperature was kept at 60 to 240 °C. The mass spectra were the mass range *m*/*z* 35–450.^17^

GC analyses were performed by using an Agilent 6890N GC system. The FID detector temperature was set to 300 °C. Simultaneous autoinjection was done to obtain equivalent retention times. Relative percentages (%) of the volatiles were calculated using FID chromatograms. This process was performed by MassFinder 4 Library, GC/MS Library, in-house “Baser Library of Essential Oil Constituents” by analyzing either authentic samples or the relative retention index (RRI) of *n*-alkanes.^18,19^

### 3.4 LOX Enzyme Inhibitory Activity

The *in vitro* 5-LOX (1.13.11.12, 7.9 units/mg) enzyme inhibition assay was performed with the colorimetric method.^20^ The % inhibition was calculated as the absorbance change per minute of enzyme activity compared to the absorbance change per minute of the tested oils and compounds. Nordihydroguaiaretic acid (NDGA) was used as a positive control. The analyses were performed in four replicates, and the results are given as mean and standard deviation (SD) have recently being reported.
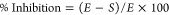
where *E* is the absorbance of the enzyme without the sample and *S* is the absorbance of the enzyme with the test sample.

### 3.5 Statistical Analysis

Statistical analysis was carried out using GraphPad Prism, ver. 7.02 (La Jolla, California, USA). *In vitro* data was expressed as mean ± standard deviation (mean ± SD). The enzyme activity was accepted as statistically significant (*p* < 0.05).

Using the rescaled distances in the dendrogram and a cutoff point (Euclidean distance) that allows the attainment of consistent clusters, the number of clusters was computed. To determine the similarity between the EO and the fractions regarding the contents of their chemical compositions, HCA was utilized (Minitab 19, State College, PA, USA).^21^

Additionally, to recognize volatile components of EO and the fractions, a Venn analysis was carried out.^22^
